# *Bacillus subtilis* extracellular protease production incurs a context‐dependent cost

**DOI:** 10.1111/mmi.15110

**Published:** 2023-06-28

**Authors:** Thibault Rosazza, Lukas Eigentler, Chris Earl, Fordyce A. Davidson, Nicola R. Stanley‐Wall

**Affiliations:** ^1^ Division of Molecular Microbiology, School of Life Science University of Dundee Dundee UK; ^2^ Mathematics, School of Science and Engineering University of Dundee Dundee UK; ^3^ Present address: Evolutionary Biology Department Universität Bielefeld Konsequenz 45 Bielefeld 33615 Germany

**Keywords:** extracellular proteases, nutrient accessibility, public good dilemma

## Abstract

Microbes encounter a wide range of polymeric nutrient sources in various environmental settings, which require processing to facilitate growth. *Bacillus subtilis*, a bacterium found in the rhizosphere and broader soil environment, is highly adaptable and resilient due to its ability to utilise diverse sources of carbon and nitrogen. Here, we explore the role of extracellular proteases in supporting growth and assess the cost associated with their production. We provide evidence of the essentiality of extracellular proteases when *B. subtilis* is provided with an abundant, but polymeric nutrient source and demonstrate the extracellular proteases as a shared public good that can operate over a distance. We show that *B. subtilis* is subjected to a public good dilemma, specifically in the context of growth sustained by the digestion of a polymeric food source. Furthermore, using mathematical simulations, we uncover that this selectively enforced dilemma is driven by the *relative* cost of producing the public good. Collectively, our findings reveal how bacteria can survive in environments that vary in terms of immediate nutrient accessibility and the consequent impact on the population composition. These findings enhance our fundamental understanding of how bacteria respond to diverse environments, which has importance to contexts ranging from survival in the soil to infection and pathogenesis scenarios.

## Graphical abstract

*Bacillus subtilis* uses extracellular proteases to access nutrients contained in polymeric sources. The extracellular proteases are a public good and support the growth of non‐producers. The cells producing the proteases are subject to a context‐dependent cost that triggers the public good dilemma.

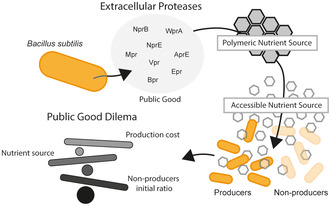

## 1 INTRODUCTION

In the natural environment, there is the potential for bacteria to encounter a wide range of environmental conditions that differ in terms of nutrient availability and accessibility levels (Brooks et al., 2011; DeLong & Pace, 2001). In contrast, laboratory experiments often use standardised levels of readily accessible nutrients that frequently do not reflect the physiology of the natural environments in which bacteria species reside (Palkova, 2004). Soil habitats are one such example of an environment that can be associated with demanding growth conditions in which nutrient diversity, abundance and accessibility vary across both macro and micro scales (Turner, 2021). Several species of soil‐associated bacteria are promising candidates for sustainable alternatives to fertilisers used in commercial agriculture (Gomez‐Godinez et al., 2021). Therefore, understanding bacterial growth dynamics in demanding environmental conditions will advance their development and efficacy.

One mechanism that microbes use to process complex, polymeric nutrient sources is the secretion of enzymes. The suite of enzymes required is linked to the ecological niche occupied by the producing strain (Henke et al., 2011; Huang et al., 2022; Ramin & Allison, 2019). For example, *Vibrio cholerae* produces chitinase to break down chitin into oligosaccharides, which supports growth (Drescher et al., 2014). In *Pseudomonas aeruginosa*, quorum sensing and other stationary phase regulators control the production of extracellular proteases that have a role in virulence (Loarca et al., 2019; Sandoz et al., 2007) and growth on polymeric nutrient sources (Li & Lee, 2019). Another example is the *Trichoderma reesei* cellulases, which are involved in depolymerising plant cell wall polysaccharides to access carbon (Martinez et al., 2008). In addition to the diversity of secreted enzymes that are encoded across different species, it has been shown that individual cells within bacterial communities can be metabolically heterogeneous (Evans et al., 2020). For example, spatially resolved single‐cell transcriptomics has revealed that in *P. aeruginosa* extracellular enzymes are produced in subpopulations of cells within an isogenic community (Dar et al., 2021). Furthermore, these enzymes are secreted products and thus are prone to exploitation by non‐producers (cheaters) and are considered a ‘public good’ (Smith & Schuster, 2019). Exploring public good production dynamics is tightly linked with exploring the cost of production and possible exploitation. Social dynamics can lead to the occurrence of a *public good dilemma*; exploitation of the public good by non‐producers leads to an increase in their relative density and consequently to a reduction in public good abundance and eventually to population collapse (Smith et al., 2019).

*Bacillus subtilis* is a Gram‐positive bacterium that synthesises several known or proposed classes of public goods that are linked to plant root colonisation (Gallegos‐Monterrosa et al., 2016) and its plant growth‐enhancing properties (Arkhipova et al., 2005). *B. subtilis* has been isolated from a broad range of environmental conditions, including many different soil environments, and the isolates exhibit high phenotypic variability (Kalamara et al., 2021). This phenotypic variability reveals an extensive ability to adapt, which is in part due to its capability to grow using a broad range of carbon and nitrogen sources (Oh et al., 2007; Polonca, 2020). However, little is known about how this bacterium grows efficiently under environmental conditions that do not allow direct metabolisation of nutrients. Despite this gap in knowledge, *B. subtilis* is currently commercialised and extensively used as a biofertiliser (Mahapatra et al., 2022) and is included as a bioactive element in other formulations including probiotics and household surface cleaners (Lee et al., 2019).

*Bacillus subtilis* produces a group of eight extracellular proteases (Schonbichler et al., 2020), which mediate the extracellular degradation of proteins (Harwood & Kikuchi, 2022) and have been postulated to be a public good required to support growth (Gray et al., 2019). Transcriptional regulation of extracellular protease production is intricate and largely tied to nutrient stress via CodY (Brantl & Licht, 2010) and entry into stationary phase via the response regulator DegU (Murray et al., 2009). Significant heterogeneity of transcription of at least some of the extracellular protease coding regions in isogenic populations is apparent (Veening et al., 2008). Consistent with complex regulatory pathways controlling their production, there is no single regulator that can be deleted which completely ablates extracellular protease production and, in some cases, post‐translational proteolytic cleavage modulates extracellular protease activity (Harwood & Kikuchi, 2022). In this study, we provide evidence to support the longstanding conjecture that extracellular proteases produced by *B. subtilis* play a critical role in facilitating access to carbon and nitrogen contained within exogenous polymeric sources. Through a comparison of wild‐type *B. subtilis* strain (NCIB 3610, WT) with an isogenic mutant lacking all eight genes responsible for extracellular protease production (Δ8), we demonstrate the selective essentiality of these enzymes for growth and reveal their public good property. By integrating mathematical modelling with experimental approaches, we predicted and assessed the impact of sharing this resource within a bacterial community containing varying proportions of extracellular enzyme producing and non‐producing cells in different nutrient contexts. Our results reveal that *B. subtilis* incurs a context‐dependent cost of producing extracellular proteases that significantly impacts population composition and productivity. These findings shed new light on the importance of extracellular proteases in bacterial survival and provide valuable insights for understanding population dynamics and productivity in diverse environmental contexts.

## 2 RESULTS

### 2.1 Extracellular proteases are essential for growth when nutrients are in polymeric form

**FIGURE 1:**
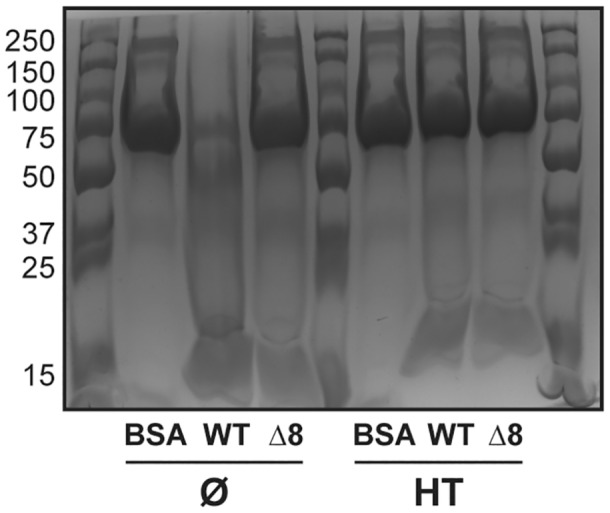
Selection of a polymeric nutrient source. BSA digestion assay using the culture supernatant after monoculture culture of NCIB 3610 (WT) and NRS5645 (Δ8) strains before (Ø) and after heat treatment (HT). BSA protein was mixed in water (BSA) or with culture supernatants for 12 h at 37°C. A representative image of three independent experiments is shown. BSA, bovine serum albumin.

**FIGURE 2:**
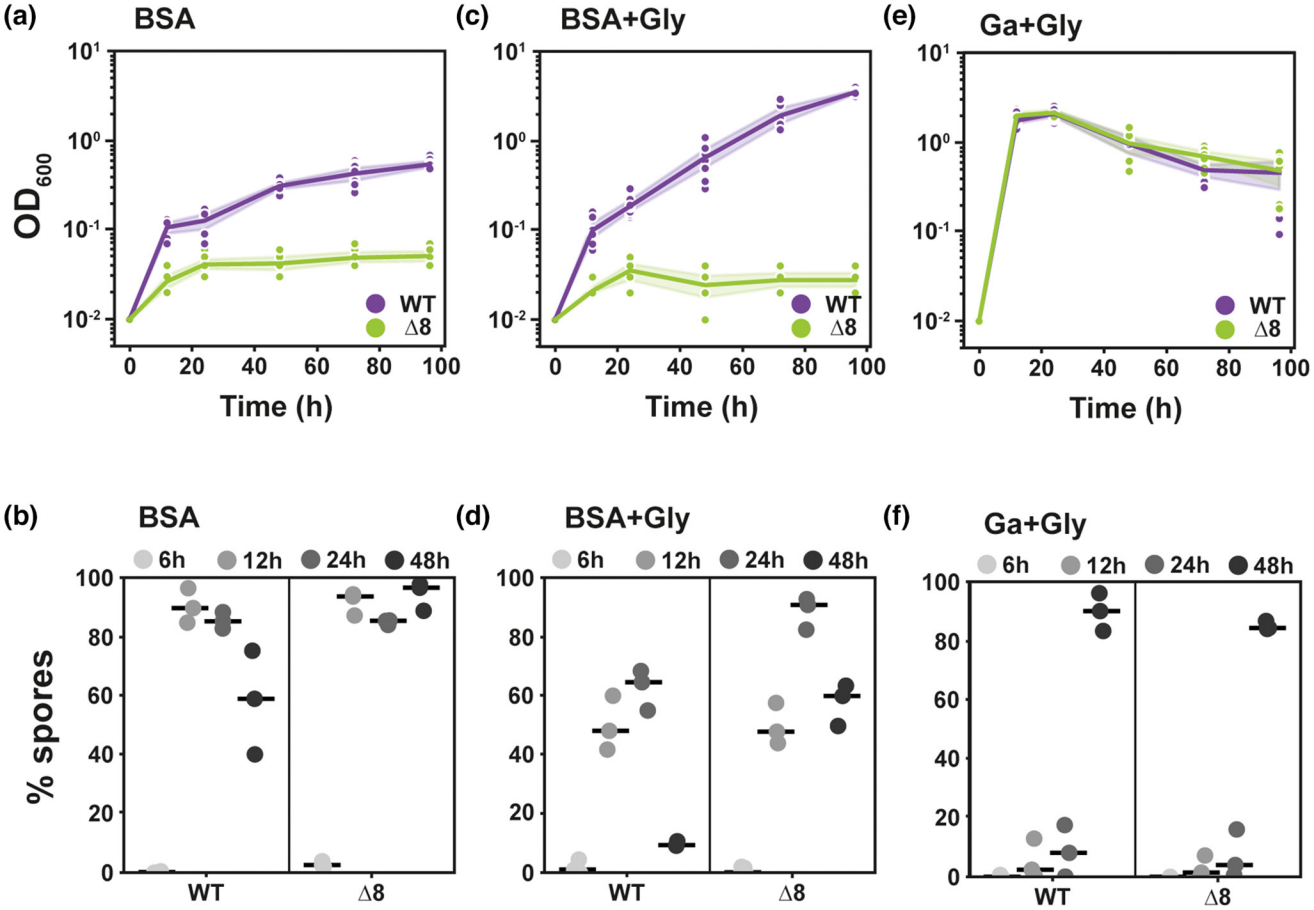
Extracellular proteases are essential for growth when using polymeric nutrient sources. Growth (a, c, e) and percentage sporulation (b, d, f) of NCIB 3610 (WT, purple) and NRS5645 (Δ8, green) in (a, b) 1% BSA (w/v) (BSA); (c, d) 0.5% glycerol (v/v) and 1% BSA (w/v) (BSA + gly); (e, f) 0.5% glutamic acid (w/v) and 0.5% glycerol (v/v) (Ga + Gly). (a, c, e) Points represent OD_600_ values (*n* = 3 biological replicates with two technical replicates), lines represent median and coloured areas represent CI 95%. (b, d, f) Points represent % spores (*n* = 3 biological replicates) and lines represent median. BSA, bovine serum albumin.

To explore the essentiality of the extracellular proteases when *B. subtilis* is faced with a polymeric nutrient source, we first selected a suitable protein to use as the nutrient source. We chose bovine serum albumin (BSA) and showed its suitability by demonstrating the ability of the extracellular proteases produced by *B. subtilis* NCIB 3610 to digest it (Figure 1). We used the extracellular protease mutant, which lacks the *aprE*, *bpr*, *epr*, *mpr nprB*, *nprE*, vpr and wprA (hereafter ‘Δ8’) as a control (Figure 1). We followed growth of the WT and Δ8 strains and quantified the proportion of spores when the cells were provided with BSA as the sole source of carbon and nitrogen. In these conditions, only growth of the WT strain was observed (Figure 2a) and most of the proportion of its population was in spore form (Figure 2b). The Δ8 strain was not able to grow and over 90% of the population sporulated and remained in that state for the duration of the experiment (Figure 2a; Figure S1b). When we added glycerol (an additional carbon source) to the culture medium, we again observed that only the WT strain was able to grow (Figure 2c; Figure S1a). We additionally note that the yield attained was higher than that reached in the presence of BSA alone. We observed that, as before, a considerable proportion of the population of both the WT and Δ8 strains formed heat‐resistant spores by 12 h, but in contrast, the level of spores reduced in the WT strain by 48 h (Figure 2d). These data are consistent with the observed increase in growth. When extracellular proteases are not needed to access nutrients in the growth medium (upon provision of glutamic acid and glycerol as the nutrient sources), both the WT and Δ8 strains displayed a rapid exponential phase before entering stationary phase (Figure 2e; Figure S1b) and have the same profile of sporulation over time (Figure 2f). Collectively, our results prove the long‐held conjecture that the extracellular proteases have a role in feeding *B. subtilis* via the degradation of proteins.

### 2.2 Extracellular protease production is unresponsive to environmental conditions

**FIGURE 3:**
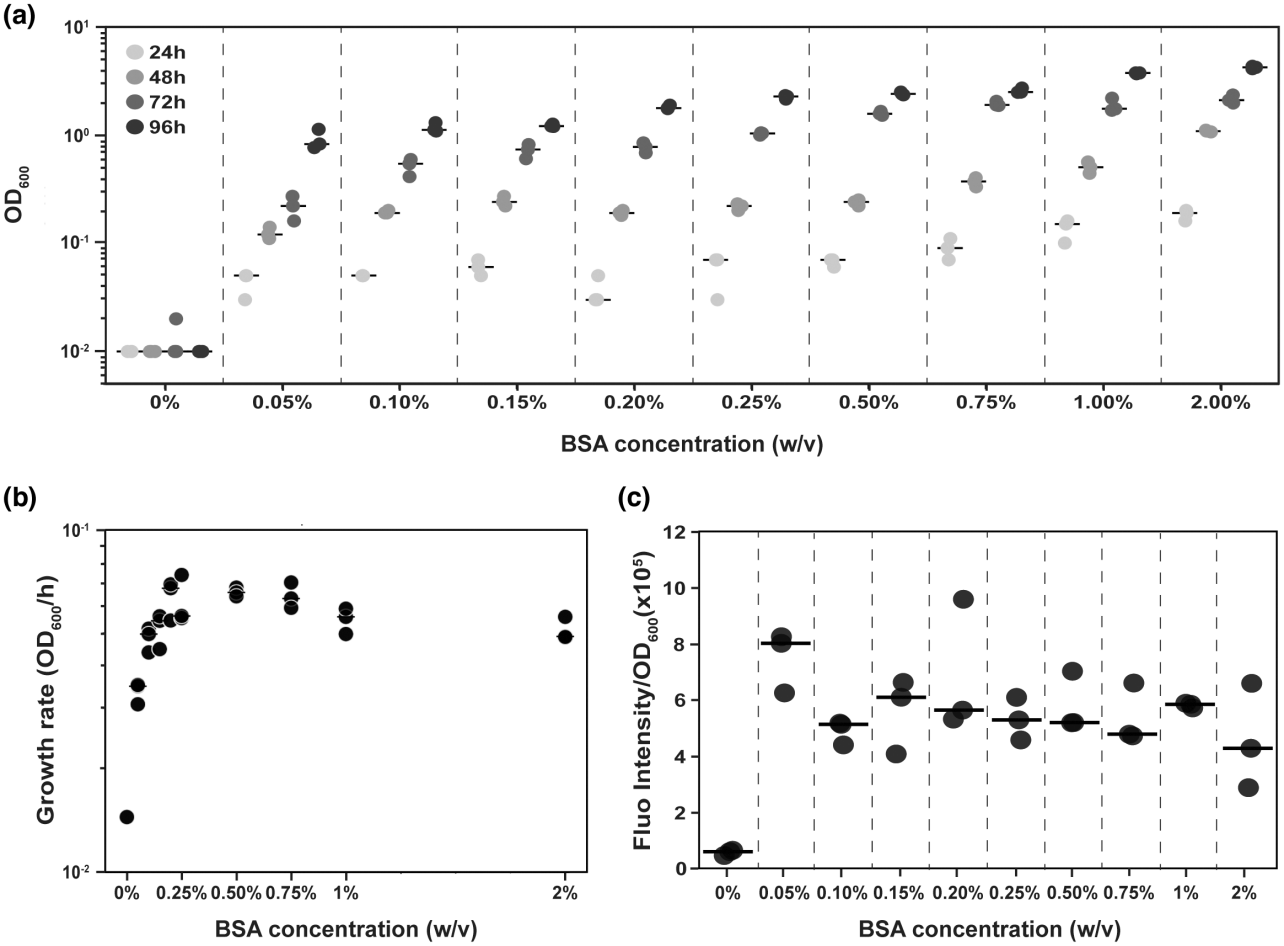
Extracellular protease production is not influenced by the nutrient levels. (a) Yield of the NCIB 3610 strain obtained at different time points in media containing 0.5% glycerol (v/v) with a BSA concentration ranging between 0% and 2% (w/v). Points represent OD_600_ values (*n* = 3) and lines represent the median. (b) The growth rate obtained from exponential regression performed on the yield of growth values displayed in (a). Each point represents the growth rate calculated for each replicate (*n* = 3) for all BSA concentration ranging between 0% and 2% (w/v). The lines represent the median. (c) Extracellular protease activity in the supernatant normalised to the total yield for a BSA concentrations ranging between 0% and 2% (w/v). Points represent fluorescence intensity/OD_600_ (*n* = 3), and lines represent the median. BSA, bovine serum albumin.

We hypothesised that if *B. subtilis* was responsive to the nutrient conditions, it would alter the level of extracellular proteases produced in accordance with the culture conditions. Therefore, we explored if there was an impact on growth and extracellular protease production by the WT strain when the BSA concentration varied over a range of 0.05% to 2% (w/v). (We used 0.5% [v/v] glycerol as an additional carbon source in all cases to enhance growth.) Analysis of the exponential growth phase across each BSA concentration (Figure 3a) revealed a saturation effect with broadly comparable growth rates after the BSA concentration exceeded 0.25% (w/v) (Figure 3b). Below this threshold, the rate of growth decreased with decreasing BSA concentration (Figure 3b). We compared the level of extracellular protease activity in the spent culture supernatant of the WT strain after growth at 48 h. In all cases, the extracellular protease activity levels were comparable after normalisation to the yield (Figure 3c). It is important to note that the extracellular proteases are stable in the culture supernatant for at least 24 h (Figure S2a). Therefore, the values we measured represent the pool of extracellular proteases that had accumulated over time. As a control, we ensured that the presence of BSA in the growth medium did not interfere with the extracellular protease quantification process (Figure S2b). Collectively, our data identify a two‐phase response when *B. subtilis* is grown using a polymeric nutrient source. When nutrients levels are low, it is these that limit growth, as there are proteases available to degrade polymeric nutrients in the medium. Thus, increasing the level of nutrients results in an increase in exponential phase growth rate. However, when nutrients are abundant, then it is the extracellular proteases that are the limiting factor as further addition of nutrients does not increase extracellular protease production.

### 2.3 Extracellular protease collective action is needed to attain full growth potential

**FIGURE 4:**
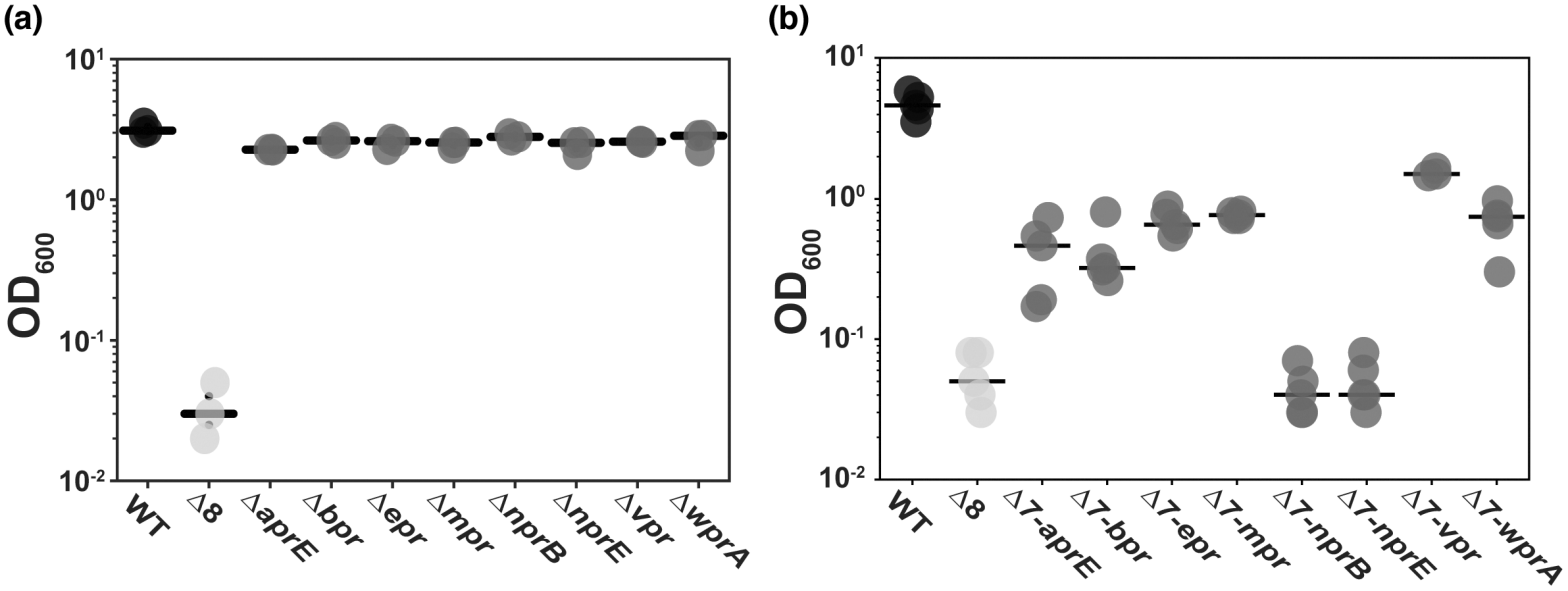
Collective role of extracellular proteases in supporting growth. Yield at 96 h in media containing 0.5% (v/v) glycerol and 1% (w/v) BSA for NCIB 3610 (WT), NRS5645 (Δ8) and for (a) single deletions strains *ΔaprE*, *Δbpr*, *Δepr*, *Δmpr*, *ΔnprB*, *ΔnprE*, *Δvpr*, *ΔwprA* (NRS3657, NRS6047, NRS3658, NRS3659, NRS3660, NRS3661, NRS7010 and NRS6810, respectively) and for (b) single extracellular protease producer strains *Δ7‐aprE*, *Δ7‐bpr*, *Δ7‐epr*, *Δ7‐mpr*, *Δ7‐nprB*, *Δ7‐nprE*, *Δ7‐vpr*, *Δ7‐wprA* (NRS3679, NRS3680, NRS3681, NRS 3882, NRS3683, NRS3684, NRS3685 and NRS6362, respectively). In both cases each point represents the OD_600_ values (*n* = 3 biological replicates and 1 technical replicate) and the lines represent the median. BSA, bovine serum albumin.

The analysis so far reveals the collective impact of deleting all eight genes encoding the extracellular proteases from the genome of NCIB 3610. We next explored the impact on growth when (i) each coding region was deleted individually (Table 1) and (ii) when each extracellular protease coding region was individually reintroduced into its native locus on the Δ8 genome (Table 1; Figure S3a). We measured growth attained by each strain after 96 h (as before in the presence of glycerol and BSA) and observed that there was a limited impact of deleting any single extracellular protease encoding gene, with only a modest, but consistent, reduction in growth yield at 96 h observed (Figure 4a). In contrast, individually returning the coding regions for *aprE*, *bpr*, *epr*, *mpr*, *vpr* or *wprA* into the Δ8 strain allowed for a partial recovery of growth when using BSA as the sole nitrogen source (Figure 4b). There was no recovery of growth when the *nprB* or *nprE* coding regions were reintroduced to the genome (Figure 4b). The ability of the spent culture supernatant harvested from the monoproducer strains to digest BSA was tested; only the *mpr* single producer strain showed any visible, albeit partial, digestion of BSA (Figure S3b). These data highlight that collective action of the extracellular proteases is required to fully support feeding of *B. subtilis* on polymeric materials.

**TABLE 1:** Strains used in this study.

Strain	Genotype^a^	Source/construction^b^
NCIB 3610	Prototroph	*Bacillus* Genetic Stock Centre
NRS6017	NCIB 3610 *comI* ^*Q12L*^	Konkol et al. (2013)
NRS5645 (called Δ8)	NCIB 3610 *comI* ^*Q12L*^ Δ*aprE*, *Δbpr*, *Δepr*, *Δmpr*, *ΔnprB*, *ΔnprE*, *Δvpr*, *ΔwprA*	Earl et al. (2020)
NRS1473	NCIB 3610 *sacA*‐P*spachy‐gfp* (*kan*)	Verhamme et al. (2007)
NRS6556	NCIB 3610 *comI* ^Q12L^ *amyE:: Phy‐spank‐mKate2* (*cml*)	Gift from Professor Akos T. Kovacs
NRS6932	NCIB 3610 *sacA*‐P*spachy‐bfp* (*kan*)	Eigentler et al. (2021)
NRS3648	Δ8 *amyE:: Phy‐spank‐gfpmut2* (*cml*)	pBL165 → NRS5645
NRS3670	Δ8 *amyE:: Phy‐spank‐mKate2* (*cml*)	pNW725 → NRS5645
NRS3656	Δ8 *amyE:: Phy‐spank –mTagBFP* (*spec*)	pNW2304 → NRS5645
BKE10300	168 Δ*aprE::erm*	Koo et al. (2017)
BKE38400	168 Δ*epr::erm*	Koo et al. (2017)
BKE02240	168 Δ*mpr::erm*	Koo et al. (2017)
BKE11100	168 Δ*nprB::erm*	Koo et al. (2017)
BKE14700	168 Δ*nprE::erm*	Koo et al. (2017)
NRS3657	NCIB 3610 *comI* ^Q12L^ Δ*aprE::erm*	BKE10300 → NRS6017
NRS6047	NCIB 3610 *comI* ^Q12L^ Δ*bpr*	Earl et al. (2020)
NRS3658	NCIB 3610 *comI* ^Q12L^ Δ*epr::erm*	BKE38400 → NRS6017
NRS3659	NCIB 3610 *comI* ^Q12L^ Δ*mpr::erm*	BKE02240 → NRS6017
NRS3660	NCIB 3610 *comI* ^Q12L^ Δ*nprB::erm*	BKE11100 → NRS6017
NRS3661	NCIB 3610 *comI* ^Q12L^ Δ*nprE::erm*	BKE14700 → NRS6017
NRS7010	NCIB 3610 *comI* ^Q12L^ Δ*vpr*	Earl et al. (2020)
NRS6810	NCIB 3610 *comI* ^Q12L^ Δ*wprA*	Earl et al. (2020)
NRS3671	NCIB 3610 *comI* ^Q12L^ *aprE* ^*WT*^ (*erm*)	pNW2600 → NRS6017
NRS3672	NCIB 3610 *comI* ^Q12L^ *bpr* ^*WT*^ (*erm*)	pNW2601 → NRS6017
NRS3673	NCIB 3610 *comI* ^Q12L^ *epr* ^*WT*^ (*erm*)	pNW2602 → NRS6017
NRS3674	NCIB 3610 *comI* ^Q12L^ *mpr* ^*WT*^ (*erm*)	pNW2603 → NRS6017
NRS3675	NCIB 3610 *comI* ^Q12L^ *nprB* ^*WT*^ (*erm*)	pNW2604 → NRS6017
NRS3676	NCIB 3610 *comI* ^Q12L^ *nprE* ^*WT*^ (*erm*)	pNW2605 → NRS6017
NRS3677	NCIB 3610 *comI* ^Q12L^ *sacA::kan vpr* ^*WT*^	*pSac‐Kan* (Middleton & Hofmeister, 2004) → NRS6017
NRS3679	NCIB 3610 Δ7 *aprE* ^*WT*^ (*erm*)	NRS3671 → NRS5645
NRS3680	NCIB 3610 Δ7 *bpr* ^*WT*^ (*erm*)	NRS3672 → NRS5645
NRS3681	NCIB 3610 Δ7 *epr* ^*WT*^ (*erm*)	NRS3673 → NRS5645
NRS3682	NCIB 3610 Δ7 *mpr* ^*WT*^ (*erm*)	NRS3674 → NRS5645
NRS3683	NCIB 3610 Δ7 *nprB* ^*WT*^ (*erm*)	NRS3675 → NRS5645
NRS3684	NCIB 3610 Δ7 *nprE* ^*WT*^ (*erm*)	NRS3676 → NRS5645
NRS3885	NCIB 3610 Δ7 *sacA::kan vpr* ^*WT*^	NRS3677 → NRS5645
NRS6362	NCIB 3610 Δ7 *wprA* ^*WT*^	Earl et al. (2020)

^a^ Drug resistance cassettes are as follows: *kan*, kanamycin resistance; *cml*, chloramphenicol resistance; *spec*, spectinomycin resistance; and *erm*, erythromycin resistance.

^b^ Strain construction is denoted as DNA from donor strain transformed into recipient strain following the direction of the arrow (→). The reference is provided if the strain has previously been described. pNW and pDR numbers refer to plasmids (see Table S1) and BKE numbers refer to strains obtained from the BKE library (Koo et al., 2017).

### 2.4 Extracellular proteases are a public good

**FIGURE 5:**
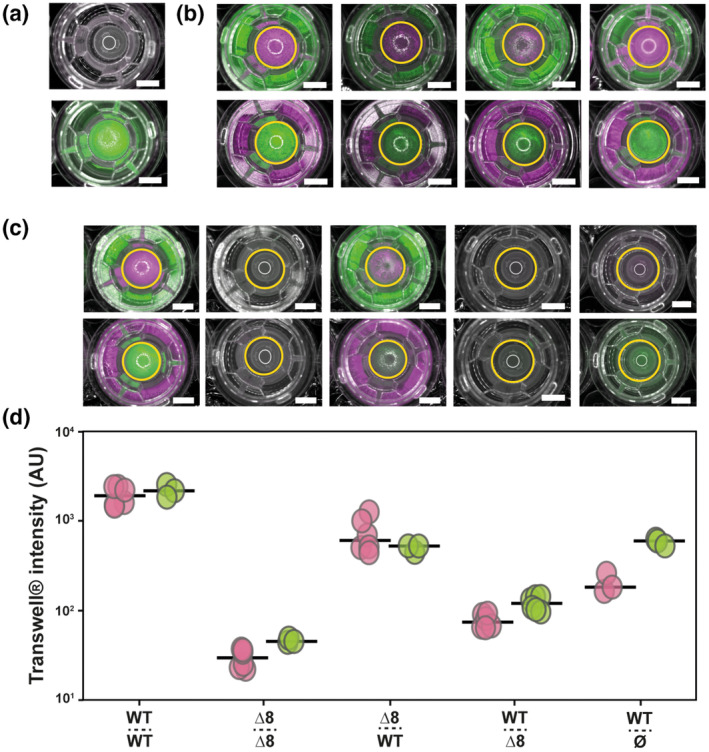
Extracellular proteases are a public good. (a) Transwell® assay control merge images of reflected light and GFP (false coloured green) fluorescence channel after growth in 0.5% glutamic acid (w/v) and 0.5% glycerol (v/v) (right) and WT‐GFP (NRS1473) (green) only inoculated in the inner Transwell (left). (b) Transwell assay merge images of reflected light, fluorescence from both the GFP (false coloured green) and mKate (false coloured magenta) channels after growth in 0.5% glutamic acid (w/v) and 0.5% glycerol (v/v) media for various strain combinations. WT‐GFP (NRS1473) or WT mKate (NRS6932) and Δ8 GFP (NRS3648) or Δ8 mKate (NRS3670). Conditions are annotated following this pattern: strain above dashed‐line = Transwell population, name below dashed‐line = outer well population. The yellow circles represent the region of interest used to quantify fluorescence values in the Transwell. For (a, b) a representative image of three independent experiments is shown. The scale bar represents 5 mm. (c) Transwell assay merge images of reflected light, fluorescence from both the GFP (false coloured green) and mKate (false coloured magenta) channels after growth in 1% BSA (w/v) and 0.5% glycerol (v/v) media for different strain combinations [see (b)]. A representative image of three independent experiments is shown. The scale bar represents 5 mm. (d) Transwell fluorescence intensity after growth as detailed in (c). Each point represents fluorescence intensity values (*n* = 3 biological replicates with two technical replicates) and the line represents the median value. BSA, bovine serum albumin.

We next tested if the extracellular proteases are a public good. We used a Transwell® system to physically separate WT and Δ8 strains within a shared, stationary growth environment but on that allows for diffusion of proteins and other molecules through a 0.4 mm pore. To allow quantification of growth in these conditions, we genetically modified the strains such that they constitutively produced either mKate2 or GFP (Table 1). We initially performed control experiments to assess that there was no cell diffusion between the Transwells® and the wells of the plates (Figure 5a). Next, to ensure that the growth of *B. subtilis* was compatible with the Transwell system, we used the nutrient media in which both the WT and Δ8 strains can grow (recall Figure 2e; Figure S1b). We observed growth of the strains in both the upper and lower chambers of the Transwell, irrespective of the strain inoculation combination used (Figure 5b; Figure S4).

When the Transwell culture conditions forced a dependency on the extracellular proteases for growth, as expected, the WT‐WT strain pairings grew efficiently, and no growth was observed for the Δ8‐Δ8 strain pairings (Figure 5c,d). For the Δ8‐WT strain combination, we observed robust growth of both strains when the Δ8 strain was within the smaller Transwell and the WT in the large outer well. However, when the Δ8 strain was within the large outer well and the WT was in the small upper Transwell no growth was measured for either strain. When the WT was in the small upper Transwell, and no cells were added to the outer well, a 5‐fold (±2.6 SD) increase in growth of the WT was quantified compared to the conditions when the Δ8 strain occupied the large outer well. These results allow us to make two conclusions: (i) extracellular proteases can act *distally* and, therefore, can be considered as a *public good*; the extracellular protease‐producing NCIB 3610 strain can facilitate the growth of the extracellular protease‐non‐producing strain at a distance and (ii) the coculture can experience a *public good dilemma*; when the WT was limited to the smaller upper well *and* the Δ8 was in the larger outer well, neither strain could grow indicating this configuration initiates an unsustainable balance of producer and non‐producer to the ultimate detriment of both strains.

### 2.5 The extracellular protease public good dilemma is context‐dependent

**FIGURE 6:**
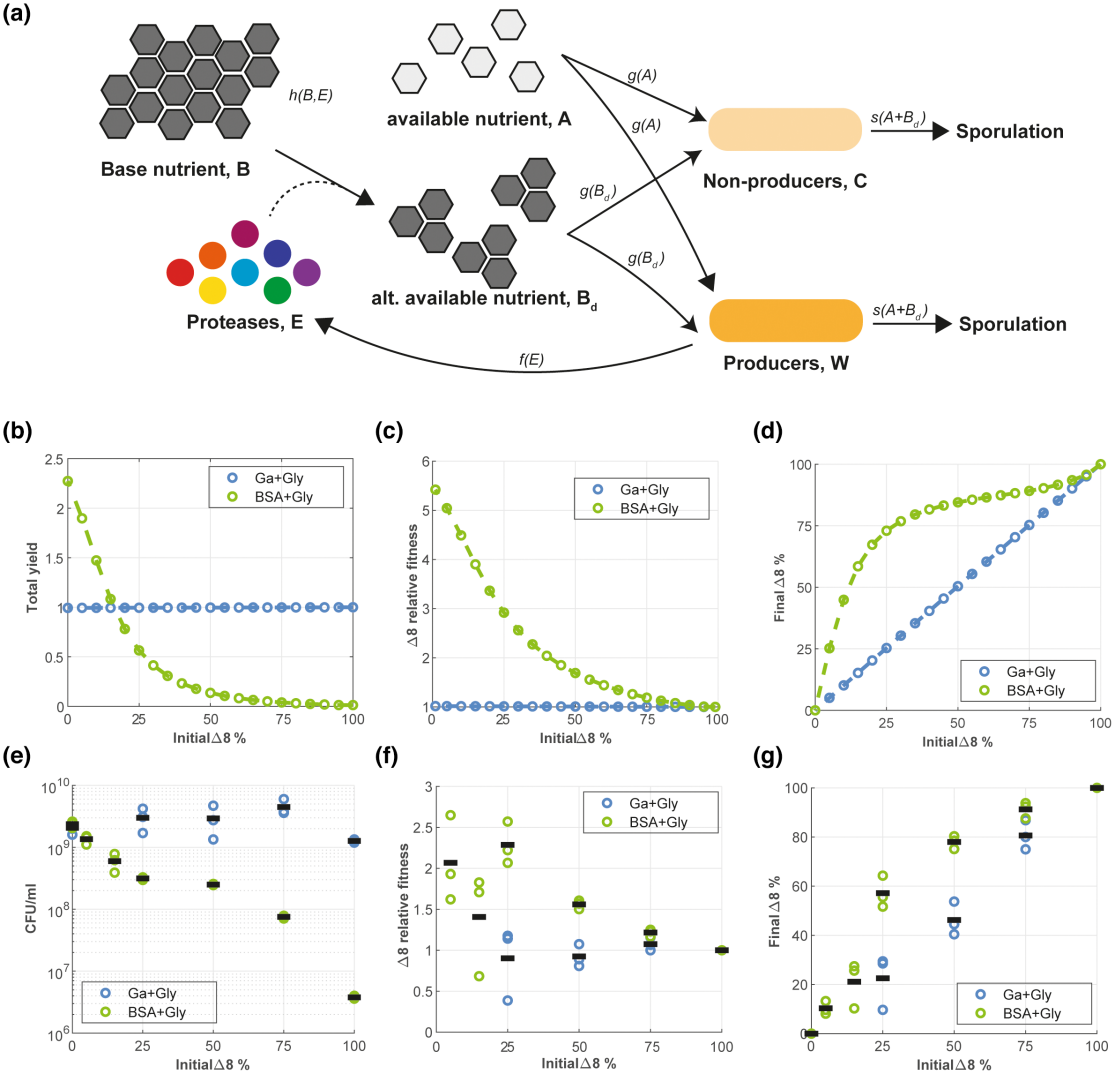
Exploration of the public good dilemma when extracellular proteases are shared. (a) Schematic of the mathematical model. Producers, *W*, of extracellular proteases *E* and non‐producers *C*, can grow in response to two different nutrient sources; a nutrient *A*, representing glutamic acid/glycerol, and an alternative nutrient *B*_*d*_, representing degraded BSA. Both producers and non‐producers are assumed to sporulate in response to a lack of nutrient. Available nutrients are represented by A in the model. BSA is represented by B. The nutrient A can be used directly by W and C. However, B requires the action of the protease E to convert it to $B_{d}$ and before it can be used by W or C. (b) *In‐silico* total yield ($W+C+W_{s}+C_{s}$) at t=100 for different initial strain proportions. (c) *In‐silico* non‐producer relative fitness $RF_{C}$ at t=100 for changing initial strain proportions. (d) *In‐silico* non‐producer proportion (% $C+C_{s}$) at t=100 for changing initial strain proportions. (e) CFU/mL representing the total population of WT‐GFP (NRS1473) and Δ8‐BFP (NRS3656) coculture in glutamic acid and glycerol (GA + Gly) (blue) and BSA and glycerol (BSA + Gly) (green) for different initial ratio of WT:Δ8 inoculum ranging from 0:100 to 100:0. Each point represents the total CFU/mL (*n* = 3) and the lines represent the median. (f) Relative fitness advantage of Δ8‐BFP (NRS3656) population compared to initial Δ8‐BFP (NRS3656) population from WT‐GFP (NRS1473) and Δ8‐BFP (NRS3656) coculture in glutamic acid and glycerol (GA + Gly) (blue) and BSA and glycerol (BSA + Gly) (green) for different initial ratio of WT:Δ8 inoculum ranging from 0:100 to 100:0. Each point represents the calculated relative fitness (*n* = 3), and the lines represent the median. (g) Final proportion of Δ8‐BFP (NRS3656) population compared to initial Δ8‐BFP (NRS3656) population from WT‐GFP (NRS1473) and Δ8‐BFP (NRS3656) coculture in glutamic acid and glycerol (GA + Gly) (blue) and BSA and glycerol (BSA + Gly) (green) for different initial ratio of WT: Δ8 inoculum ranging from 0:100 to 100:0. Each point represents the total CFU/mL (*n* = 3), and the lines represent the median. BSA, bovine serum albumin.

We used a mathematical framework to further explore the potential occurrence of a public good dilemma in the context of extracellular protease production by *B. subtilis*. We devised a continuum framework comprising ordinary differential equations that describe the growth of wild‐type cells $W\left(t\right)$ and Δ8 (‘non‐producer’) cells $C\left(t\right)$ over time t≥0. We assumed growth was within a well‐mixed environment. Cells were assumed to grow (no extracellular proteases required) in response to an ‘available nutrient’ $A\left(t\right)$ that represented glutamic acid/glycerol. It was assumed that BSA, represented by $B\left(t\right)$, could not be directly consumed by cells. However, $B\left(t\right)$ was assumed to be degraded by extracellular proteases, $E\left(t\right)$ into a degraded form of BSA represented by $B_{d}\left(t\right).$ It was assumed that cells could grow in response to $B_{d}\left(t\right)$, but with a nutrient‐to‐biomass conversion rate less than that associated with glutamic acid $A\left(t\right)$ (Figure 6a). We tested the model for single‐strain cultures across different simulated nutrient conditions [namely, those representing (i) glutamic acid and glycerol and, (ii) BSA and glycerol] through appropriate choice of initial nutrient abundances. We found strong agreement between the growth of single‐strain cultures *in‐silico* and in experimental assays across all the conditions (compare Figure S5 and Figure 2).

Next, we employed the model to investigate the potential outcomes of co‐culturing the WT and Δ8 strains over a wide range of initial strain ratios. We defined the *in‐silico yield* to be the *total* biomass density predicted by the model and includes both vegetative cells and spores as measured at the end of each simulation. For *in‐silico* growth conditions representing the medium containing non‐polymeric nutrient sources (glutamic acid and glycerol), the model predicted no change to the total yield as the initial proportion of Δ8 varied (Figure 6b). However, for *in‐silico* growth conditions representing the medium containing polymeric nutrients, we observed a significant impact on total yield when changing the initial proportion of Δ8 (Figure 6b). For example, the introduction of 10% Δ8 cells into a WT population led to a 35% decrease in total yield compared to a single‐strain WT culture. Moreover, for an initial Δ8 ratio of 50%, total yield is reduced by a factor of 10, and for ratios above ~75%, the total yield essentially saturates to zero. Hence, proportions of WT less than ~25% are predicted to be incapable of supporting a dominant non‐producing strain with resultant collapse of both strains (recall Figure 5).

We also used our mathematical model to predict the relative abundance of both extracellular protease producers (WT) and non‐producers (Δ8). The model predicts that if the extracellular proteases are not required for growth, the non‐producing Δ8 strain does not gain a fitness advantage (Figure 6c). Hence, the final relative abundance of Δ8 is set by its initial proportion (Figure 6d). By contrast, in the case where extracellular proteases are required for growth, as expected, the relative fitness advantage of the Δ8 strain was highest when it was rare in the initial population (Figure 6c). Moreover and because of this advantage, the relative abundance of Δ8 increased from its initial proportion across all values (Figure 6d). However, the model revealed that the greatest *absolute* increase in the percentage Δ8 population was predicted to occur when the initial population contained ~25% Δ8 strain. This matches the ratio that induces a significant relaxation in total yield (reduced by ~75%) (Figure 6b,d).

Motivated by the model predictions, we experimentally analysed bacterial cultures grown with different WT to Δ8 starting ratios. Consistent with our *in‐silico* results, the findings show that sharing extracellular proteases with non‐producing cells induced a reduction in total yield only when the extracellular proteases are needed to support growth (Figure 6e). Moreover, this reduction effect was found to respond in a non‐linear manner to increasing the initial fraction of Δ8, with broadly the same saturating profile as that predicted by the model (cf. Figure 6b,e noting the log scale in Figure 6e). Inspection of the proportion of the two strains in the final culture revealed that if extracellular proteases were not required for growth, the Δ8 strain did not have a fitness advantage (Figure 6f) and the relative population proportions remained constant (Figure 6g). However, in the case that extracellular proteases were required for growth, a fitness advantage was afforded to the Δ8 strain (Figure 6f) and the introduction of an initially small proportion of the Δ8 strain led to an increased final Δ8 strain fraction (Figure 6g). Again, the ‘sweet spot’ from the Δ8 perspective, in terms of its ability to maximise absolute proportional increase, appeared to occur at the 25% initial ratio data point. Collectively these data demonstrate the growth advantage gained by the Δ8 strain is dependent on the growth medium and the initial population composition.

### 2.6 The relative cost of extracellular protease production underpins the public good dilemma

**FIGURE 7:**
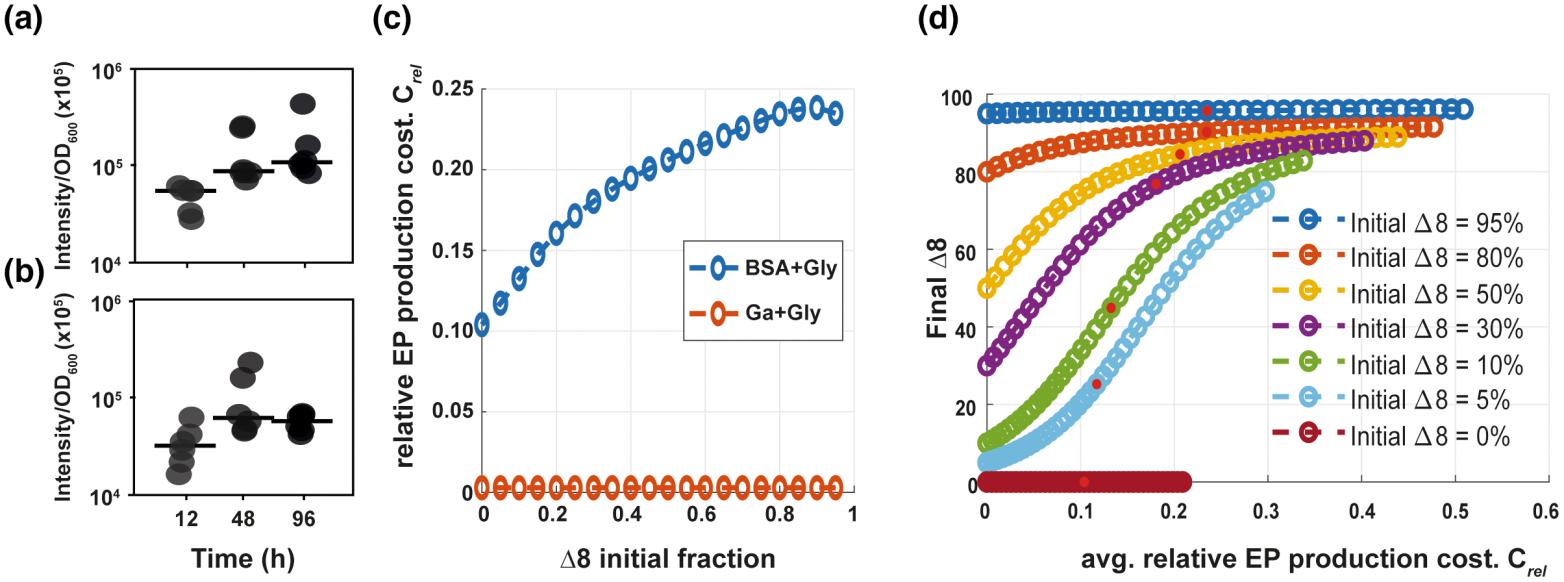
Relative cost of extracellular protease production explains the public good dilemma. (a) Growth of NCIB 3610 in planktonic culture at 12 h for glutamic acid 0.5% (w/v) and 0.5% glycerol (v/v) (Ga + Gly) and 48 h for 1% BSA (w/v) and 0.5% glycerol (v/v) (BSA + Gly). Points represent OD_600_ values (*n* = 3 biological replicates with two technical replicates), lines represent median. (b) Extracellular protease activity in the supernatant of culture from (a) normalised to the total yield represented in (a). Points represent fluorescence intensity/OD_600_ (*n* = 3 biological replicates with two technical replicates), and lines represent the median. (c) Relative cost of extracellular protease production against the initial fraction of Δ8 in growth media representing glutamic acid and BSA as the nitrogen sources. (d) *In‐silico* proportion of the non‐producer at t=100 against the *in‐silico* relative cost of extracellular protease production during growth in a medium containing BSA as the sole source of nitrogen. Note that both axes represent model outputs. Data points correspond are generated by varying the value of χ (0≤χ≤2). Red dots represent $\chi_{s}=1$, the parameter value used in other simulations (see Table 2).

We have revealed the existence of a public good dilemma associated with the production of extracellular proteases that manifests only when these proteases are required for growth. This presents as a contradiction, because irrespective of the media choice, the WT population maintained similar levels of extracellular protease production when normalised to yield (Figure 7a,b), with the same metabolic (*absolute*) cost per extracellular protease unit. Therefore, we hypothesised that the selective penalty on growth incurred by the WT strain may be a result of the *relative cost* of extracellular protease production, that is the *ratio* of the growth penalty associated with extracellular protease production relative to total growth. We hypothesised that this relative cost may be different in each of the media contexts. We tested this hypothesis by solving the model and comparing the computed *relative cost* in both media contexts across a range of initial WT to Δ8 population ratios, initially for a fixed absolute cost (χ).

The model simulations revealed that in conditions where the growth medium contains non‐polymeric nutrients (glutamic acid and glycerol), the relative cost of extracellular protease production is small (<0.01) for all initial Δ8 proportions (Figure 7c). Recall that the WT and Δ8 strains performed equally well, with the final ratios closely matching their corresponding initial values (Figure 6c). Thus, the model confirms that for non‐polymeric growth media, a low relative cost correlates with the WT and Δ8 growing equivalently, irrespective of the initial Δ8 proportion. By contrast, when simulated in conditions representing the use of polymeric nutrient sources, the relative cost of extracellular protease production was computed to be at least an order of magnitude higher, across all initial Δ8 proportions and to increase with the Δ8 fraction (Figure 7c). We tested the robustness of our predictions by varying the absolute cost (parameter χ) from a hypothetical case of zero absolute cost $\left(\chi =0\right)$, to twice the value used in all other simulations $\left(\chi =2\chi_{s}\right)$. In simulated non‐polymeric nutrient conditions, the relative cost remained small despite the range of values of the absolute cost. Additionally, the final ratio of the Δ8 strain remained essentially unaltered from its initial value (Figure S6). By contrast, in the simulated polymeric nutrient source medium, increasing χ over the same range caused the relative cost to increase significantly for each initial Δ8 ratio (Figure 7d). Moreover, as the relative cost for production increased, the final Δ8 fraction also increased from its initial value (Figure 7d). For each initial ratio, the greatest increase in the final Δ8 proportion occurred at the highest relative cost value.

Combined, our simulations predict the public good dilemma to be a context (nutrient source) dependent phenomenon. We demonstrated that for a fixed value of the absolute cost associated with extracellular protease production, the relative cost in each simulated media differed by at least an order of magnitude: when using non‐polymeric nutrient sources, the relative cost for extracellular protease production is small, whereas in media requiring extracellular proteases for growth the relative cost is at least an order of magnitude higher. It is this high *relative* cost that determines a growth advantage to the non‐producers in this context and the higher this relative cost, the greater that advantage.

## 3 DISCUSSION

Here we reveal that when growth of *B. subtilis* is dependent on the activity of extracellular proteases, the extracellular protease‐producing cells incur a significant cost of sharing this resource, and the total yield of the whole community is reduced because of a public good dilemma. By deploying a combination of molecular genetics, physiological assays and mathematical modelling, we provide evidence supporting the long‐held, but previously unsubstantiated conjecture that the extracellular proteases of *B. subtilis* are a public good that supports growth via the degradation of exogenous proteins to release nutrients, as has previously been shown to be the case for *P. aeruginosa* despite the fact that the suite of proteases is regulated using a different mechanism (Li & Lee, 2019). We established that in environmental conditions in which cells require extracellular proteases to grow, more than one of the extracellular proteases in the suite produced by the bacterium is required. Production of any single extracellular protease is insufficient to attain growth levels comparable to that of the wild‐type strain and removal of any single extracellular protease coding region can be predominantly counteracted by the production of the remaining suite of proteases. Finally, we reveal that a low density of cells can generate enough extracellular protease activity to support growth. This survival tactic is distinct from coordination of extracellular protease production by quorum sensing during infection, which requires a cell density threshold to be exceeded (Lyczak et al., 2000). Moreover, as *B. subtilis* uses small peptides to monitor cell density during quorum sensing (Kalamara et al., 2018), it is unclear if quorum sensing pathways are activated when extracellular proteases are essential for growth given the role of extracellular proteases in producing quorum sensing peptides (Lanigan‐Gerdes et al., 2007), or if any released quorum sensing peptides are rapidly used as a nutrient source.

### 3.1 Dependence on extracellular proteases for growth

*Bacillus subtilis* produces a suite of eight extracellular proteases, some of which have been shown to have specific molecular targets and roles (Connelly et al., 2004; Corvey et al., 2003; Earl et al., 2020; Harwood & Kikuchi, 2022; Kobayashi & Ikemoto, 2019). Additionally, the extracellular proteases had been proposed to have a role in supporting growth, based on their ability to digest polymeric molecules (El Mayda et al., 1986) and by analogy with the mode of action of other enzymes, including chitinase and alginate lyase (Drescher et al., 2014; Ebrahimi et al., 2019). However, for *B. subtilis*, while the involvement of the extracellular proteases in sustaining survival in deep starvation (oligotrophic) conditions has been shown (Gray et al., 2019), a focus has been placed on the ability of *B. subtilis* to sporulate under nutrient limitation conditions. Explicit evidence supporting the role of extracellular proteases in feeding when nutrients are abundant, but in a polymeric form, has been lacking. The construction of an extracellular protease‐producing deletion strain, in an otherwise prototrophic strain background, allowed us to gain new insights into the growth‐supporting role of extracellular proteases. We revealed the role exerted by each of the extracellular proteases in isolation through the generation of a suite of monoproducer strains (Gray et al., 2019; Zhao et al., 2019). We identified that individual reintroduction of six of the eight known extracellular protease coding regions allowed some recovery of growth. Interestingly, the Mpr monoproducing strain showed partial digestion of BSA and a partial recovery of growth, this is even though Mpr has been shown to be activated upon cleavage by Bpr (Park et al., 2004). These results suggest that extracellular proteases may be self‐activated or show limited activity, in specific contexts, although further investigation is needed to fully understand their mode and extent of activation.

We also explored how *B. subtilis* balances its ability to sporulate with its ability to use extracellular proteases to sustain growth when presented with an abundant, but polymeric nutrient source. We uncovered that when presented with such a nutrient source, part of the population immediately sporulates. However, part of the population remains active and produces extracellular proteases that release usable nutrients from the polymeric source. Consistent with heterogeneous expression of the extracellular protease genes in the population (Veening et al., 2008) we assume that the release of nutrients subsequently allows the spores to germinate and growth of the population to occur. It will be of interest to explore the dynamics of sporulation and extracellular protease production in more depth in conditions where propagation of the cells is dependent on the activity of the enzymes to establish the timing of each process with respect to feedback from the local environment.

### 3.2 The extracellular protease public good dilemma is context‐dependent

*Bacillus subtilis*, like many other microbes, produces an array of public goods including specialised metabolites with antimicrobial activity (Caulier et al., 2019) and structural components of the biofilm extracellular matrix (Charlton et al., 2022; Hobley et al., 2013; Otto et al., 2020). The cost incurred by the cells when producing public goods is variable and can be context‐dependent (Kraigher et al., 2021; Sexton & Schuster, 2017; Waite & Shou, 2012). Moreover, the extent to which public goods can be shared within and between populations varies (Dragos et al., 2018; Lyons & Kolter, 2017, 2018). Here we highlight the public good attributes of *B. subtilis* extracellular proteases, uncovering that either the activity of the extracellular proteases, or the nutrients released after their action, can be shared with physically separated, non‐producing cells within the same growth environment. We also established that the ‘public good dilemma’ is only triggered in certain nutrient conditions, namely those in which nutrients are in polymeric form and growth is, therefore, dependent on the extracellular proteases. In these conditions, the producer cells incur a significant penalty on growth and the non‐producing cells are afforded a fitness advantage. However, there is no measurable penalty to the wild‐type strain when it produces similar a level of extracellular proteases in conditions when they are not required for growth; there was no difference in growth yield to that observed for the Δ8 strain. These outcomes are like those previously identified for *P. aeruginosa* where accessing polymeric nutrients relies on extracellular proteases and there is a cost to the population which produces them (Smith & Schuster, 2019). Indeed, cells can acquire a single mutation in the genome (e.g. in quorum sensing systems) that eliminates production of this suite of enzymes and acquire a fitness advantage (Robinson et al., 2020). The context‐selective cost contrasts markedly with other public goods produced by *B. subtilis*, for example the biofilm exopolysaccharide (Dragos et al., 2018) where a significant growth advantage is afforded to non‐producing cells (Jautzus et al., 2022) irrespective of whether the matrix molecule provides an advantage or not. As production of extracellular proteases and biofilm matrix molecules are both heterogeneous within an isogenic population of cells, it will be of interest to learn how the different relative costs are balanced, if there is reciprocal sharing of the different classes of public good and the impact on structuring across divergent nutrient conditions.

Mathematical modelling allowed us to deduce that it is the *relative* cost of extracellular protease production that underpins the context dependency of the public good dilemma. When extracellular proteases are not required to support growth, the relative cost associated with their production is negligible. However, the slow‐down of growth when cells use polymeric substrates as the source of nutrients increased the relative cost to a level that significantly impacted the growth rate. Thus, the total yield of both wild‐type and non‐producing cells was reduced. It may be that the public good dilemma is amplified by the fact that at least some of the genes involved in extracellular protease production are heterogeneously expressed (Marlow et al., 2014; Veening et al., 2008), meaning that even the WT extracellular enzyme producing population will already contain non‐producers. Overall, our results highlight the importance of the nutrient landscape in triggering a public good dilemma (Drescher et al., 2014; Sexton & Schuster, 2017), a situation that could significantly affect the development of a bacterial community (Granato et al., 2019; Palmer & Foster, 2022).

### 3.3 Outlook

*Bacillus subtilis* is a soil‐dwelling bacterium that has been shown to live on both decaying plant matter (Earl et al., 2008; Siala et al., 1974) and on living plant roots (Blake et al., 2021). Therefore, it is highly likely that extracellular proteases support growth in the bacterium's natural environment by accessing polymeric nutrients released during decay and growth and/or contained within the soil itself (Rillig et al., 2007; Schonbichler et al., 2020). As *B. subtilis* has promising applications as a biofertiliser (Mahapatra et al., 2022; Sun et al., 2020), it is important to understand how *B. subtilis* can settle and survive in diverse environmental conditions. We believe our findings will provide a foundation on which to build an understanding of how *B. subtilis* can survive in environments that diverge significantly in terms of nutrient accessibility.

## 4 EXPERIMENTAL PROCEDURES

### 4.1 Strain construction

Strains used in this study were derived from *B. subtilis* isolate NCIB 3610 or NCIB 3610 *comI* ^*Q12L*^ (stocked here as NRS6017) (Konkol et al., 2013) (Table 1). To prepare competent cells, we used a media containing 60 mM K_2_HPO_4_, 37 mM KH_2_PO_4_, 95 mM d‐Glucose, 3 mM sodium citrate dihydrate, 10 mM l‐glutamic acid monopotassium salt, 0.1% (w/v) casein enzymatic hydrolysate, 3 mM MgSO_4_, 0.8 mM FeCl_3_ (Konkol et al., 2013). A single colony was grown in 2 mL at 37°C, 200 rpm for 4.5 h. Then, 400 μL of this culture was mixed with 20 μL of gDNA (ranging from 100 ng μL^−1^ to 1 μg μL^−1^) and incubated for 1.5 h at 37°C, 200 rpm. 100–200 μL of the sample was plated onto an LB plate (1% [w/v] tryptone, 0.5% [w/v] yeast extract, 1% [w/v] NaCl and select agar 1.5% [w/v]) with antibiotic for selection. Extracellular protease monoproducer strains were constructed by the insertion of an erythromycin resistance gene proximal to the wild‐type coding region in NRS6017, followed by the transfer of the allele to the Δ8 genome using the antibiotic selection marker. The introduction of the single extracellular protease coding region in the native location on the genome was assessed by PCR. See Table S1 for all primer sequences used in this study and Table S2 for all plasmids used in this study.

### 4.2 Genome sequencing and analysis

Genome sequencing was provided by MicrobesNG (http://www.microbesng.uk). For sample preparation, single colonies of each strain to be sequenced were resuspended in sterile phosphate‐buffered saline (PBS) buffer (137 mM NaCl, 2.7 mM KCl, 10 mM Na_2_HPO_4_, 1.8 mM KH_2_PO_4_ pH 7.4) and streaked onto LB agar plates. The plates were incubated at 37°C overnight and the following day. For short read sequencing, genomic DNA was extracted using QIAGEN kit (69504; QIAGEN) and resuspended in EB buffer. For enhanced genome sequencing, the cells were harvested, placed into the barcoded bead tubes provided and sent to the MicrobesNG facilities. There, for each sample, three beads were washed with extraction buffer containing lysozyme and RNase A, incubated for 25 min at 37°C. Proteinase K and RNaseA were added and incubated for 5 min at 65°C. Genomic DNA was purified using an equal volume of SPRI beads and resuspended in EB buffer. DNA was quantified in triplicate with the Quantit dsDNA HS assay in an Eppendorf AF2200 plate reader. Genomic DNA libraries were prepared using Nextera XT Library Prep Kit (Illumina) following the manufacturer's protocol with the following modifications: two nanograms of DNA instead of one were used as input, and PCR elongation time was increased to 1 min from 30 s. DNA quantification and library preparation were carried out on a Hamilton Microlab STAR automated liquid handling system. Pooled libraries were quantified using the Kapa Biosystems Library Quantification Kit for Illumina on a Roche light cycler 96 qPCR machine. Libraries were sequenced on the Illumina HiSeq using a 250 bp paired end protocol. Reads were adapter trimmed using Trimmomatic 0.30 with a sliding window quality cut‐off of Q15 (Bolger et al., 2014). De novo assembly was performed on samples using SPAdes version 3.7 (Bankevich et al., 2012), and contigs were annotated using Prokka 1.11 (Seemann, 2014). Annotated draft assemblies of the sequencing results were acquired, whole genome sequencing data were visualised in Artemis software (Rutherford et al., 2000) and mutation predictions were determined using Breseq (Deatherage & Barrick, 2014).

### 4.3 Preparing cells for culture and assessing growth

Material from a −80°C glycerol stock of the required strains was streaked onto an LB plate and incubated O/N at 30°C. A single colony was used to inoculate 5 mL of LB and incubated O/N at 200 rpm at 37°C. 100 μL of the O/N culture was added to 5 mL of LB and incubated at 200 rpm at 37°C. After 4 h, the culture was centrifuged for 10 min at 4500 rpm. The cell pellet was resuspended using 1 mL of base MS media (5 mM K_2_HPO_4_, 5 mM KH_2_PO_4_, 100 mM MOPS pH 7.0) and OD_600_ was measured. The culture density was normalised to an OD_600_ of 1 by adding base MS media as required. In 50 mL Corning® tubes, 5 mL of base MS media was supplemented with metal mix (2 mM MgCl_2_, 700 μM CaCl_2_, 50 μM MnCl_2_, 50 μM FeCl_3_, 1 μM ZnCl_2_, 2 μM thiamine) and inoculated to an OD_600_ of 0.01. The growth media also contained 0.5% (w/v) glutamic acid, 0.5% (v/v) glycerol or BSA at between 0.05% and 2% (w/v) as required. Note that the growth medium containing glycerol and glutamic acid is referred to as MSgg and has been used for a wide array of biofilm studies (Branda et al., 2001). Here we use it as a defined growth medium. The samples were incubated at 200 rpm at 37°C. The OD_600_ of the cultures was measured after 12, 24, 48, 72 or 96 h incubation as indicated. An aliquot of the culture was sampled for spore or protease activity quantification. A separate culture tube was used for each time point.

### 4.4 Measuring the percentage of spores

At 6, 12, 24 and 48 h the cultures were collected and diluted at a 1/10 ratio in 1× PBS. Serial dilutions were plated onto LB agar plates to provide the total CFU/mL. The serially diluted samples were heat‐treated for 20 min at 80°C followed by 20 min at room temperature. The remaining CFU/mL (representing spores) was calculated after growth on agar plates incubated O/N at 37°C.

### 4.5 Extracellular proteases activity quantification

To measure the level of extracellular proteases a 1 mL sample from a planktonic culture was centrifuged for 10 min at 10,000 rpm (the OD_600_ was measured). The supernatant was recovered and used with the protease fluorescent detection kit (PF0100; Sigma‐Aldrich). The protocol was adjusted from the manufacturers' instructions in the following ways: (i) the samples were allowed to incubate for 4 h and (ii) 20 μL of the trichloroacetic acid precipitation supernatant was used instead of 2 μL. The fluorescent signal at 485 nm was acquired using a 96 black well plate (Corning; CLS3603‐48EA) and a PHERAstar FSX plate reader (BMG Labtech) (protocol: endpoint fluorescence intensity, 485 nm, 20 flashes, gain 100). The fluorescent signal was normalised relative to the OD_600_ of the culture.

### 4.6 BSA digestion assay

A 1 mL culture supernatant from a 24 h planktonic culture grown in media with glycerol and glutamic acid was centrifuged for 10 min at 10,000 rpm. For each sample, 500 μL of the supernatant was heat‐treated 20 min at 80°C to ablate the enzymatic activity. Fifteen microliters of the culture supernatant (plus and minus heat treatment) was mixed with 20 μg of BSA contained in 15 μL (1.33 mg/mL stock solution) and incubated at 37°C with shaking at 200 rpm for 12 h. Ten microliters of SDS loading dye (0.5 M Tris–HCl pH 6.8, 0.1 M EDTA, 15.5% [w/v] SDS, 3% [v/v] glycerol, 5% [v/v] β‐mercaptoethanol, 1 mg bromophenol blue) was added to the samples, which were heated for 5 min at 99°C. BSA integrity was assessed after separation by 10% (w/v) SDS‐PAGE, staining using Coomassie blue (ISB1L; Sigma‐Aldrich) and imaging using an Azure 600 scanner (Azure Biosystems).

### 4.7 Transwell assay

Cultures were prepared and normalised to an OD_600_ of 1. We used a Transwell with a 0.4 μM pore size (10147291; Fisher Scientific). One millilitre of cell culture was added to the larger outer well and 250 μL was added to the small upper Transwell. The samples were incubated at 30°C, with no shaking, in a plastic box (20 cm × 10 cm × 10 cm) filled with ~100 mL of water to keep the hygrometry level constant (while not submerging the plates inside the box). After 3 days (glutamic acid and glycerol) or 9 days (BSA and glycerol) of incubation, both reflected light and fluorescence signals (485 and 620 nm) were captured using a Leica M165C stereoscope. Using FIJI software, a circular area encompassing the Transwell and three circular areas within the outer well were drawn and fluorescence intensity signals were quantified, and background corrected.

### 4.8 Coculture of producers and non‐producers

Cell cultures were prepared and normalised to an OD_600_ of 1. In a volume of 1 mL of MS base two strains were mixed in a ratio of 0:100, 25:75, 50:50, 75:25, 85:15, 95:5 and 100:0 (NRS1473:NRS3656). For each condition, 5 mL of media containing glycerol and BSA was inoculated at OD_600_ of 0.01 and incubated at 200 rpm at 37°C. At 6, 12, 24 and 48 h the OD_600_ was measured, and a sample of each dilution was serially diluted in 1× PBS for CFU analysis. To capture GFP and BFP fluorescent signals and distinguish the two strains the colonies were imaged using an Azure 600 scanner (Azure Biosystems).

### 4.9 Preparing cells for culture and assessing growth

Material from a −80°C glycerol stock of the required strains was streaked onto an LB plate and incubated O/N at 30°C. A single colony was used to inoculate 5 mL of LB and incubated O/N at 200 rpm at 37°C. 100 μL of the O/N culture was added to 5 mL of LB and incubated at 200 rpm at 37°C. After 4 h, the culture was centrifuged for 10 min at 4500 rpm. The cell pellet was resuspended using 1 mL of base MS media (5 mM K_2_HPO_4_, 5 mM KH_2_PO_4_, 100 mM MOPS pH 7.0) and OD_600_ was measured. The culture density was normalised to an OD_600_ of 1 by adding base MS media as required. In 50 mL Corning tubes, 5 mL of base MS media was supplemented with metal mix (2 mM MgCl_2_, 700 μM CaCl_2_, 50 μM MnCl_2_, 50 μM FeCl_3_, 1 μM ZnCl_2_, 2 μM thiamine) and inoculated to an OD_600_ of 0.01. The growth media also contained 0.5% (w/v) glutamic acid, 0.5% (v/v) glycerol or BSA at between 0.05% and 2% (w/v) as required. Note that the growth medium containing glycerol and glutamic acid is referred to as MSgg and has been used for a wide array of biofilm studies (Branda et al., 2001). Here we use it as a defined growth medium. The samples were incubated at 200 rpm at 37°C. The OD_600_ of the cultures was measured after 12, 24, 48, 72 or 96 h incubation as indicated. An aliquot of the culture was sampled for spore or protease activity quantification. A separate culture tube was used for each time point.

### 4.10 Modelling the role of extracellular proteases

We used a continuum approach to describe the growth dynamics of bacterial cells, nutrient processing and extracellular proteases production in well‐shaken liquid cultures. Hence, we used a system of ordinary differential equations (ODEs) describing the interactions between the variables, which represent time‐dependent densities: the extracellular proteases producing strain $W\left(t\right)$ and its spores $W_{s}\left(t\right)$ that represent the wild‐type NCIB 3610; an extracellular proteases non‐producing (cheater) strain $C\left(t\right),$ and its spores $C_{s}\left(t\right)$ that represent the Δ8 mutant; an accessible nutrient $A\left(t\right)$ that represents glutamic acid; a complex nutrient source $B\left(t\right)$ that represents BSA, an intermediate nutrient $B_{d}\left(t\right)$ that represents the degraded BSA and extracellular proteases $E\left(t\right).$ Model simulations were run for times $0\leq t\leq t_{\mathrm{end}}$, where $t_{\mathrm{end}}$ represents the endpoint of our experimental assay. The system of equations used was

$$W^{\prime }={\left(\gamma_{A}g\left(A\right)+\gamma_{B_{d}}g\left(B_{d}\right)-\mathrm{\chi f}\left(E\right)\right)}_{+}W-s\left(A+B_{d}\right)W,$$

$$C^{\prime }=\left(\gamma_{A}g\left(A\right)+\gamma_{B_{d}}g\left(B_{d}\right)\right)C-s\left(A+B_{d}\right)C,$$

$$W_{s}^{\prime }=s\left(A+B_{d}\right)W,$$

$$C_{s}^{\prime }=s\left(A+B_{d}\right)C,$$

$$B^{\prime }=-h\left(B,E\right),$$

$$B_{d}^{\prime }=h\left(B,E\right)-g\left(B_{d}\right)\left(W+C\right),$$

$$A^{\prime }=-g\left(A\right)\left(W+C\right),$$

$$E^{\prime }=f\left(E\right)W,$$

where

$$\begin{array}{c} g\left(X\right)=\frac{k_{1}X^{2}}{k_{2}^{2}+X^{2}},\;s\left(X\right)=k_{3}\left(1-\frac{X^{2}}{k_{4}^{2}+X^{2}}\right),\\ \;f\left(E\right)=k_{5}\left(1-\frac{E}{k_{6}}\right),\;h\left(B,E\right)=\frac{k_{7}\mathrm{BE}}{k_{8}+B}. \end{array}$$

Both the extracellular enzyme producer W and non‐producer C were assumed to grow in response to both the accessible nutrient source A and intermediate nutrient $B_{d}$. The functions $g\left(A\right)$ and $g\left(B_{d}\right)$ describe the nutrient consumption of each nutrient, respectively, assumed to be saturating and modelled using a Hill equation with Hill coefficient 2, maximum growth rate $k_{1}>0$ and half‐saturation constant $k_{2}>0$. Corresponding growth functions ($\gamma_{A}g\left(A\right)$ and $\gamma_{B_{d}}g\left(B_{d}\right)$, respectively) were assumed to differ only through the yield coefficients $\gamma_{A}>\gamma_{B_{d}}>0$. Each unit of the producer W was assumed to produce $f\left(E\right)$ units of extracellular proteases per unit time. Due to negative feedback (the extracellular proteases degrade the quorum sensing signal ComX, which promotes extracellular protease production; Spacapan et al., 2020), the production rate $f\left(E\right)$ was assumed to be self‐limiting and decrease from its maximum $k_{5}>0$ to zero as the density E increased from zero to the carrying capacity $k_{6}>0$. The penalty on growth borne by the producer was assumed proportional to the protease production rate and given by the term $-\mathrm{\chi f}\left(E\right)W$. The parameter χ≥0 was defined to be the *absolute cost* for extracellular protease production (a measure of the metabolic cost associated with producing one unit of extracellular protease per unit producer). We noted that in some of our experiments detailed below, cells entered a late death phase, due to nutrient exhaustion. We did not consider this in the model as it did not impact on the main objective, which was to uncover what determined the growth dynamics when nutrients were present (but potentially unavailable). Hence, we assumed the growth term in the first equation is non‐negative. By definition, the non‐producer was assumed to be free of growth penalty associated with extracellular protease production. Both producers and non‐producers were assumed to sporulate at rate $s\left(A+B_{d}\right)$ in response to the total density of nutrients facilitating growth, $A+B_{d}$. The sporulation rate was assumed to decrease from its maximum $k_{3}>0$ to zero as the total nutrient density increased from zero following a Hill‐type function with Hill coefficient 2 and half‐saturation constant $k_{4}>0$. Finally, the conversion of the inaccessible base nutrient B to the intermediate nutrient $B_{d}$ induced by extracellular proteases was assumed to follow Michaelis–Menten dynamics with maximum conversion rate $k_{7}>0$ and half‐saturation constant $k_{8}>0$. The initial conditions for all cases were taken to be $W\left(0\right)=W_{0},C\left(0\right)=C_{0},W_{s}\left(0\right)=0,C_{s}\left(0\right)=0,B_{d}\left(0\right)=0,E\left(0\right)=0$. Initial conditions for B and A differed across model scenarios. We represented growth media containing glutamic acid as the main nitrogen and carbon source by $B\left(0\right)=0,A\left(0\right)=A_{0,\text{MSbg}}>0$, and growth media containing BSA as the main nitrogen source by $B\left(0\right)=B_{0,\text{MSbg}}>0,A\left(0\right)=A_{0,\text{MSbg}}>0$, where $A_{0,\text{MSbg}}$ was assumed to be much smaller than $A_{0,\text{MSgg}}$. We highlight that in this second case (growth media containing BSA as the only nitrogen source) the initial amount of accessible nutrient A was small, but non‐zero. This was to capture the observation that cells in the experimental assay displayed initial, fast growth that we concluded to be due to carryover of nutrients between the different growth conditions.

The model was numerically solved using MATLAB's ODE solver *ode15s*. Parameter values used are shown in Table 2. We were interested in qualitative agreement between the model and experimental assays and, therefore, did not estimate parameters using experimental data. However, we nevertheless ensured good qualitative fit across different nutrient conditions (see Section 2). For visualisations, we normalised computed cell‐densities using the density obtained for the in‐silico WT in stationary phase strain in the growth medium representing glutamic acid.

**TABLE 2:** Model parameter values.

Parameter	Value	Description
$\gamma_{A}$	4	Growth yield in response to nutrient A
$\gamma_{B_{d}}$	1	Growth yield in response to nutrient $B_{d}$
$\chi =\chi_{s}$	1	Exoprotease production cost per unit produced
s	0.4	Maximum sporulation rate
$k_{1}$	3	Maximum nutrient consumption rate per unit WT/D8
$k_{2}$	0.02	Nutrient uptake half‐saturation constant
$k_{3}$	0.4	Maximum sporulation rate
$k_{4}$	0.003	Sporulation half‐saturation constant
$k_{5}$	0.03	Maximum exoprotease production rate per unit WT
$k_{6}$	0.2	Exoprotease carrying capacity
$k_{7}$	0.5	Maximum nutrient conversion rate (B to B_d)
$k_{8}$	0.45	Nutrient conversion half‐saturation constant

*Note*: Parameter values used (unless otherwise stated) in all simulations of our mathematical model are shown.

### 4.11 Relative fitness

Relative fitness of a strain was calculated to be its increase in relative population size from the start of the experiment/simulation to the end point of the experiment/simulation. That is, the relative fitness of the non‐producer strain was given by

$$RF_{C}=\frac{\frac{C\left(t_{\mathrm{end}}\right)}{W\left(t_{\mathrm{end}}\right)+C\left(t_{\mathrm{end}}\right)}}{\frac{C\left(0\right)}{W\left(0\right)+C\left(0\right)}}.$$

### 4.12 The relative cost of extracellular protease production

We calculated the *relative cost* of extracellular protease production in our model as follows. First, we determined the time interval T in which the growth rate of $W\left(t\right)$ was positive, that is

$$T=\left\{t\leq t_{\mathrm{end}}:\mathrm{\chi f}\left(E\right)<\gamma_{A}g\left(A\right)+\gamma_{B_{d}}g\left(B_{d}\right)\right\}.$$

This represents the period over which wild‐type cells actively divide. We then defined the *total penalty* per unit of W of extracellular protease production during this time interval to be

$$P_{\mathrm{tot}}≔\int_{T}\mathrm{\chi f}\left(E\right)\mathrm{dt},$$

and the *total growth* per unit W in the absence of extracellular protease production to be

$$G_{\mathrm{tot}}≔\int_{T}\left(\gamma_{A}g\left(A\right)+\gamma_{B_{d}}g\left(B_{d}\right)\right)\mathrm{dt}.$$

We then defined the *relative cost* of extracellular protease production per unit W during growth as the ratio

$$C_{\mathrm{rel}}≔\frac{P_{\mathrm{tot}}}{G_{\mathrm{tot}}}.$$

It is clear from the definition of T that the relative cost $0\leq C_{\mathrm{rel}}\leq 1$.

### 4.13 Statistical and data analysis

For group comparison, ANOVA test was performed. For mean comparison over multiple conditions, Tukey's HSD test was performed. Data were analysed using Python 3.9 through Jupyter Notebook. All graphs generated were generated using Matplotlib and Seaborn packages.

## AUTHOR CONTRIBUTIONS

**Thibault Rosazza:** Conceptualization; investigation; funding acquisition; writing – original draft; methodology; validation; visualization; writing – review and editing; software; formal analysis; data curation; resources. **Lukas Eigentler:** Software; conceptualization; methodology; formal analysis; investigation; data curation; writing – original draft; writing – review and editing; visualization; supervision. **Chris Earl:** Writing – review and editing; resources; validation; methodology; conceptualization. **Fordyce A. Davidson:** Conceptualization; methodology; validation; formal analysis; resources; data curation; writing – original draft; writing – review and editing; visualization; supervision; project administration; funding acquisition. **Nicola R. Stanley‐Wall:** Supervision; writing – original draft; funding acquisition; conceptualization; visualization; writing –review and editing; formal analysis; project administration.

## CONFLICT OF INTEREST STATEMENT

The authors declare that they have no known competing financial interests or personal relationships that could have appeared to influence the work reported in this paper.

## ETHICS STATEMENT

Authors declare that no human or animal subjects were used in this study.

## Supporting information


Data S1.

## Data Availability

Computational code and experimental data sets have been deposited in the nrstanleywall GitHub repository (https://github.com/NSWlabDundee/) and archived by BioStudies (Rosazza et al., 2023a) and Zenodo (Rosazza et al., 2023b). Sequencing data sets have been deposited in the ENA portal (PRJEB59494).
