# Supplementary material for: Bacillus subtilis extracellular protease production incurs a context‐dependent cost
Source: Mol Microbiol. 2023 Jun 28;120(2):105–21. doi: 10.1111/mmi.15110 (PMC10952608; doi:10.1111/mmi.15110)
Supplement: Supplementary file 1 — Data S1. [file MMI-120-105-s001.pdf]

Supplemental Material

Bacillus subtilis extracellular protease production incurs a context-dependent cost

Thibault Rosazza[1], Lukas Eigentler[1,2,3], Chris Earl [1], Fordyce Davidson[2], Nicola Stanley-Wall[1]

[1] Division of Molecular Microbiology, School of Life Science, University of Dundee, DD1 4HN Dundee, United Kingdom

[2] Mathematics, School of Science and Engineering, University of Dundee, DD1 4HN Dundee, United Kingdom

[3] Current address: Evolutionary Biology Department, Universität Bielefeld, Konsequenz 45, 33615 Bielefeld, Germany

Corresponding authors: n.r.stanleywall@dundee.ac.uk f.a.davidson@dundee.ac.uk

Running title – The cost of *B. subtilis* extracellular protease production

Competing interest statement: The authors declare no potential competing interests

16

Primer name	Sequence 5' to 3'	Length	Tm	Nearby Gene / Reference
NSW3250	ATCCGAGCGTTGCATATGTG	20	59	aprE_1_For_RT
NSW3251	AGCCTGTGTAGCCTTGAGAG	20	59	aprE_1_Rev_RT
NSW3258	GCTGCACCGGGTTATTACAG	20	59	bpr_1_For_RT
NSW3259	ATTGGCATTCTCCGCTGTTC	20	59	bpr_1_Rev_RT
NSW3268	GGCACAGCAATCCGTATCTG	20	59	epr_2_For_RT
NSW3269	CAATGCCGTTTTGTCCTTGC	20	59	epr_2_Rev_RT
NSW3262	TGGACGGAAAGCAAAGACAC	20	59	mpr_1_For_RT
NSW3263	GGGACTGCTGCTGTTTGTAG	20	59	mpr_1_Rev_RT
NSW3272	AGCTCAAAGGGAATGCGAAC	20	59	nprB_2_For_RT
NSW3273	TGTTTTCCTTCATCGCGACC	20	59	nprB_2_Rev_RT
NSW3276	TCACCCAAGAAACAGCCAAC	20	59	nprE_2_For_RT
NSW3277	TCACCGATGTCCCAGTCTTC	20	59	nprE_2_Rev_RT
NSW3278	TTCGGCTCCTACTCTTCAGC	20	59	vpr_1_For_RT
NSW3279	ATCCTTTGCTTCGCCGATTC	20	59	vpr_1_Rev_RT
NSW3256	GTACCCTTATCAATGGCCGC	20	59	wprA_2_For_RT
NSW3257	TAAGCTTGCGTTTGGACAGC	20	59	wprA_2_Rev_RT
NSW3284	CCTTAAAACATGCAGGAATTGACG	24	67	erm_5pL (ErmR815) (Koo *et al.*, 2017)
NSW3285	ATCAAGAAATGGATCGAAGCG	21	65	aprE_3pR (BSU10300) (Koo *et al.*, 2017)
NSW3286	TTGATTTTGCTGACCAACTCC	21	64	bpr_3pR (BSU15300) (Koo *et al.*, 2017)
NSW3287	TAAAGAAGACGGGGTTTTGCT	21	64	epr_3pR (BSU38400) (Koo *et al.*, 2017)
NSW3288	CTTCAGCGGGACTGATAACAAC	22	65	mpr_3pR (BSU02240) (Koo *et al.*, 2017)
NSW3289	CTGGGCAACACCTGTAAATCC	21	66	nprB_3pR (BSU11100) (Koo *et al.*, 2017)
NSW3290	TTGATGTCGTCTGGTGATAGATG	23	64	nprE_3pR (BSU14700) (Koo *et al.*, 2017)
NSW3291	AGTGATACGATGAGGATGAGGTG	23	65	vpr_3pR (BSU38090) (Koo *et al.*, 2017)
NSW3292	ACTCGATGTAGCTGTGCAGAAG	22	64	wprA_3pR (BSU10770) (Koo *et al.*, 2017)
NSW3293	TGAAATCGGCTCAGGAAAAGG	21	59	erm_cassette_for
NSW3294	ATCGTCAATTCCTGCATGTTTT	22	57	erm_cassette_rev
NSW3295	CTGATGTCTTTGCTTGGCGA	20	59	aprE_long_for
NSW3296	CGTTTACATCGGTCTGGCTG	20	59	aprE_long_rev
NSW3297	CAAAGTGTTCCGAAGCGTGA	20	59	bpr_long_for
NSW3298	AAGCCAAATGTCACCACCAC	20	59	bpr_long_rev
NSW3299	GTGAACAACCTCCGCAATGT	20	59	epr_long_for
NSW3300	ACGGAGCATGAGAATAGGGG	20	59	epr_long_rev
NSW3301	TCTCCTCGTCCGCATGATAC	20	59	mpr_long_for
NSW3302	CGCCCCTTTTGTCTCGTAAG	20	59	mpr_long_rev
NSW3303	TACTGGCATATGGAGCAGGG	20	59	nprB_long_for

NSW3304	CCAATCAGTTCAGCCATCGG	20	59	nprB_long_rev
NSW3305	CGCAATATAACACCCGCCAA	20	59	nprE_long_for
NSW3306	TCAGGAGCGGTTTTCTGTCT	20	59	nprE_long_rev
NSW3307	ACGGCCGATTTCTTTTCTCG	20	59	vpr_long_for
NSW3308	CTTGGGGCTGTGCTTGATTT	20	59	vpr_long_rev
NSW3309	GGAGAATACGCCGGCAAAAT	20	59	wprA_long_for
NSW3310	TCTCCAACACAGCCCAATCT	20	59	wprA_long_rev

Table S1 List of primers used in this study

Plasmid name	Background vector	Insert Fragment / Reference
pBL165	pBL165	*Pspac-hy gfpmut2* (Stanley *et al.*, 2003)
pNW725	pBL165	*PtapA mKate2*
pNW2304	pDR111	*Phy-spank bfp* (Eigentler *et al.*, 2021)
pNW2600	pUC57	*aprE-erm* cassette
pNW2601	pCC1	*bpr-erm* cassette
pNW2602	pUC57	*epr-erm* cassette
pNW2603	pUC57	*mpr-erm* cassette
pNW2604	pUC57	*nprB-erm* cassette
pNW2605	pUC57	*nprE-erm* cassette
pSac-Kan	pSac	*sacA-KanR* (Middleton & Hofmeister, 2004)

Table S2 List of plasmids used in this study

Synthetic cassettes were designed using NCIB 3610 genomic DNA as a template and erythromycin (*erm*) resistance gene (Supplementary data). pSac-Kan plasmid was used for integration of the kanamycin resistance gene at the *sacA* gene.

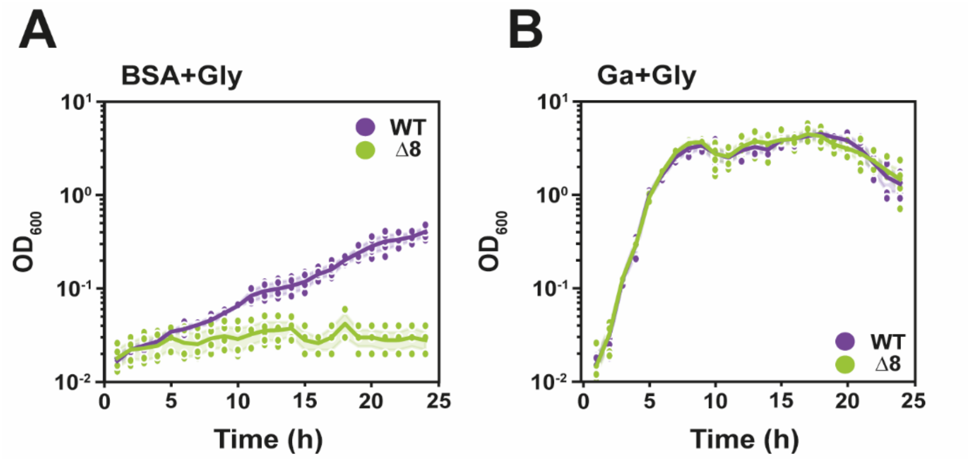

Figure S1. Growth analysis in different media. Growth curve of NCIB 3610 (WT) (purple) and NRS5645 (Δ8) (green) monoculture in (**A**) 1% BSA (w/v) and 0.5% glycerol (v/v) (BSA+Gly) and (**B**) 0.5% glutamic acid (w/v) and 0.5% glycerol (v/v) (Ga+Gly). Points represent OD_{600} values (n=3), lines represent the median and coloured-areas represent CI 95%.

.

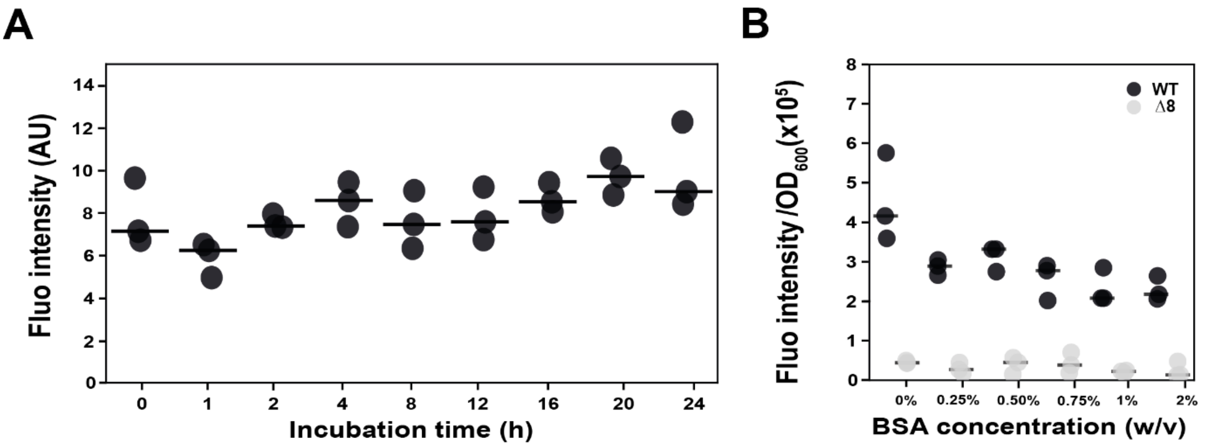

Figure S2. Extracellular protease activity assay controls. A. Extracellular proteases activity of culture supernatant obtained from NCIB 3610 monoculture in 0.5% glutamic acid (w/v) and 0.5% glycerol (v/v) (Ga+Gly) media at 24h and incubated at 37˚C over a period time ranging from 0 to 24 h. Points represent fluorescence intensity (n=3) and lines represent the median. **B.** Extracellular proteases activity of culture supernatant obtained from NCIB 3610 monoculture in 0.5% glutamic acid (w/v) and 0.5% glycerol (v/v) (Ga+Gly) media at 24h in which BSA concentration ranging from 0 to 2% (w/v) was added during the protease activity quantification assay. Points represent fluorescence intensity (n=3) and lines represent median.

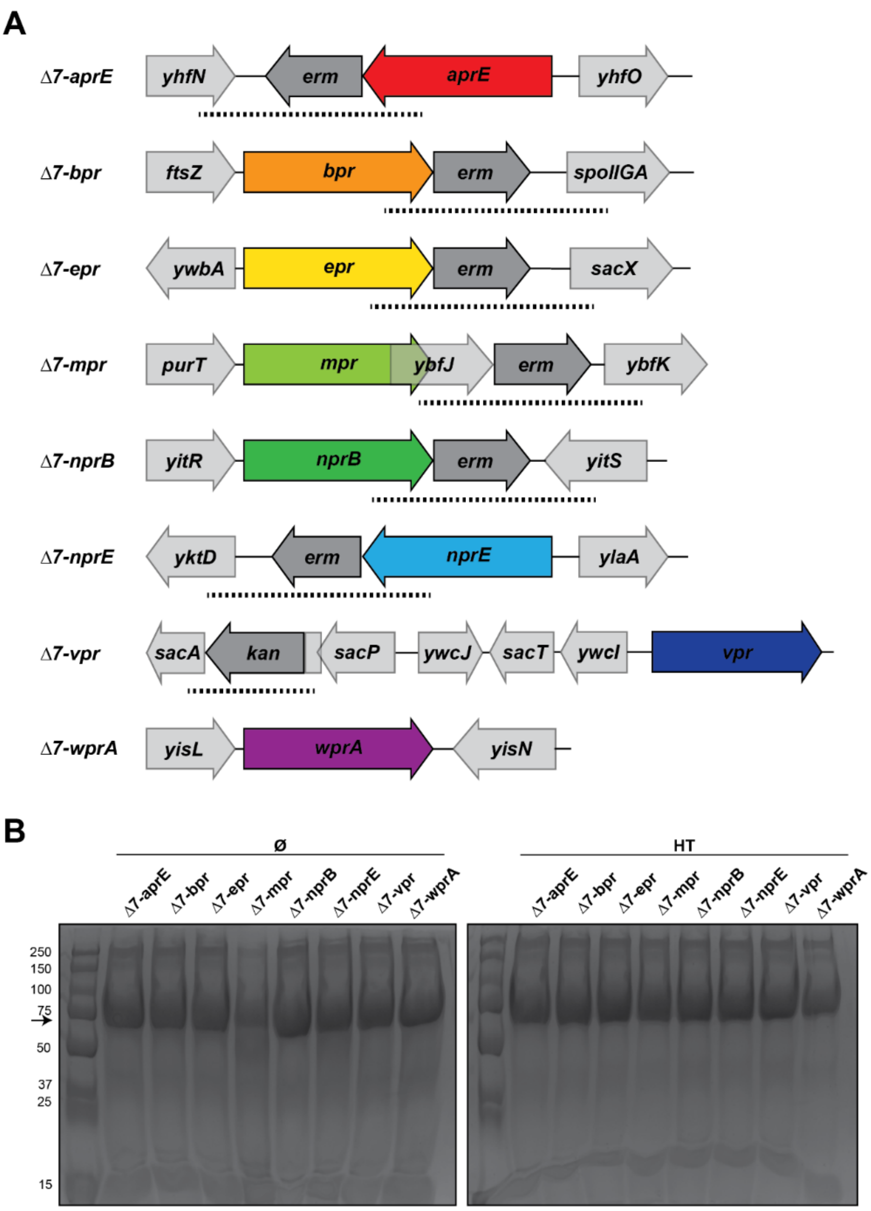

Figure S3. Monoproducer extracellular protease strains. A. Schematic of gene surrounding each extracellular protease interest for the monoproducer strains *Δ7-aprE, Δ7-bpr, Δ7-epr, Δ7-mpr, Δ7-nprB, Δ7-nprE, Δ7-vpr, Δ7-wprA* (NRS3579, NRS3680, NRS3681, NRS3682, NRS3883, NRS 3684, NRS3685, NRS6362, respectively). Dashed lines represent the region of DNA containing 500 bp of the extracellular protease gene region, erythromycin (*erm)* or kanamycin (*kan)* resistance gene and 500 bp of the downstream region of extracellular protease gene used for integration into the chromosome. **B.** BSA digestion assay using culture supernatant isolated after growth of the extracellular protease monoproducer strains *Δ7-aprE, Δ7-bpr, Δ7-epr, Δ7-mpr, Δ7-nprB, Δ7-nprE, Δ7-vpr, Δ7-wprA* (NRS3579, NRS3680, NRS3681, NRS3682, NRS3883, NRS 3684, NRS3685, NRS6362, respectively) in 0.5% glutamic acid (w/v) and 0.5% glycerol (v/v) (Ga+Gly) before (Ø) and after heat-treatment (HT). The black arrow represents BSA molecular weight (69 kDa). A representative image of three independent experiments is shown.

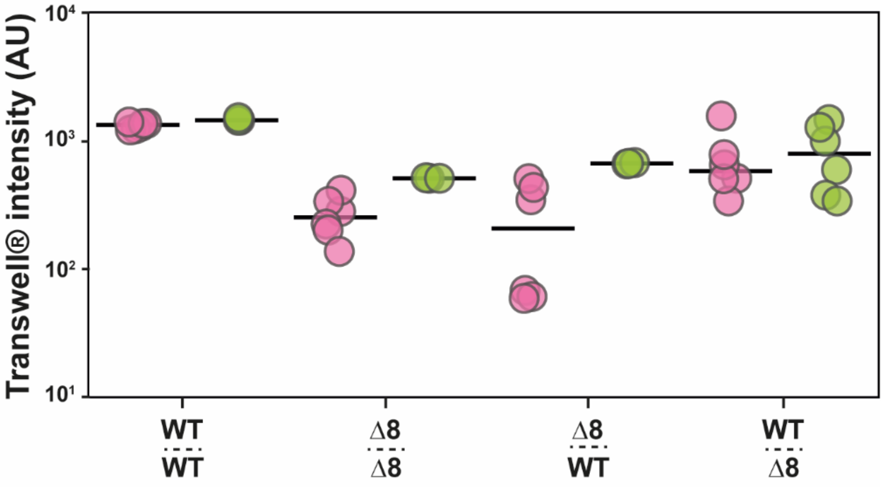

Figure S4 Quantification of growth in the Transwell®. Transwell® fluorescence intensity after growth as detailed in **Figure 5B.** Each point represents fluorescence intensity values (n=3 biological replicates with 2 technical replicates) and the line represents the median value.

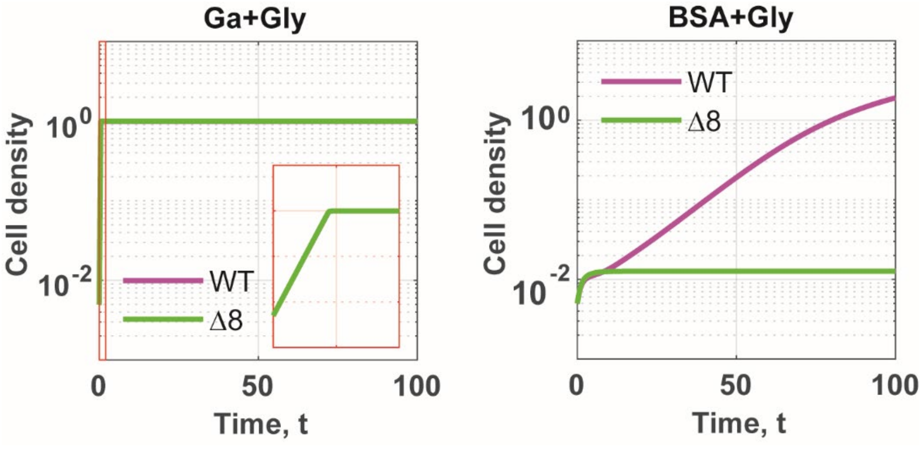

Figure S5. Supplemental *in-silico* data. Simulated single-strain cultures in different media. *In-silico* growth curves obtained from single-strain model simulations corresponding to the experimental data displayed in Fig. 2 and Fig. S1. The insets show a blow-up of the early growth dynamics. Parameter values are given in Table 2.

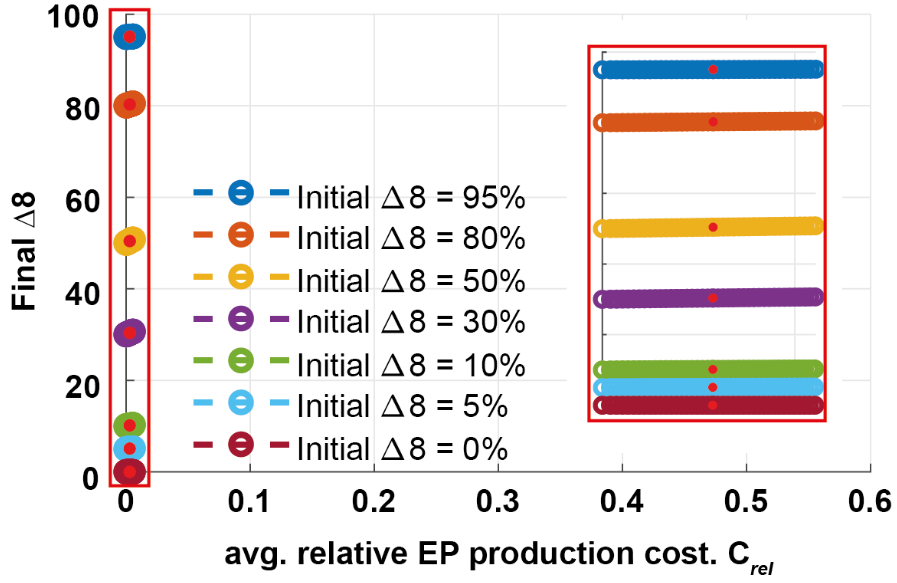

Fig S6. *In-silico* proportion of the cheater at $t = 100$ against the *in-silico* relative cost of extracellular protease production during growth in a medium containing glutamic acid as the sole source of nitrogen. Note that both axes represent model outputs. Data points correspond are generated by varying the value of χ $(0 \leq \chi \leq 2)$. Red dots represent $\chi_s = 1$, the parameter value used in other simulations (see Table 1). Note that the x axis limits are identical to those used in Fig. 7D. The inset shows the dynamics for low relative costs.

Supplemental References

Eigentler, L., Kalamara, M., Ball, G., MacPhee, C.E., Stanley-Wall, N.R., and Davidson, F.A. (2021) Founder cell configuration drives competitive outcome within colony biofilms. *bioRxiv*: 2021.2007.2008.451560.

Koo, B.M., Kritikos, G., Farelli, J.D., Todor, H., Tong, K., Kimsey, H., . . . Gross, C.A. (2017) Construction and Analysis of Two Genome-Scale Deletion Libraries for Bacillus subtilis. *Cell Syst* **4**: 291-305 e297.

Middleton, R., and Hofmeister, A. (2004) New shuttle vectors for ectopic insertion of genes into Bacillus subtilis. *Plasmid* **51**: 238-245.

Stanley, N.R., Britton, R.A., Grossman, A.D., and Lazazzera, B.A. (2003) Identification of catabolite repression as a physiological regulator of biofilm formation by Bacillus subtilis by use of DNA microarrays. *J Bacteriol* **185**: 1951-1957.
